# Capripoxviruses, leporipoxviruses, and orthopoxviruses: Occurrences of recombination

**DOI:** 10.3389/fmicb.2022.978829

**Published:** 2022-10-06

**Authors:** Alexander Sprygin, Ali Mazloum, Antoinette van Schalkwyk, Shawn Babiuk

**Affiliations:** ^1^Federal Center for Animal Health, Vladimir, Russia; ^2^Agricultural Research Council - Onderstepoort Veterinary Institute, Pretoria, South Africa; ^3^National Centre for Foreign Animal Disease, Canadian Food Inspection Agency, Winnipeg, MB, Canada

**Keywords:** poxvirus, recombination, vaccinia viruses, myxoma virus, lumpy skin disease

## Abstract

Poxviruses are double-stranded DNA viruses with several members displaying restricted host ranges. They are genetically stable with low nucleotide mutation rates compared to other viruses, due to the poxviral high-fidelity DNA polymerase. Despite the low accumulation of mutations per replication cycle, poxvirus genomes can recombine with each other to generate genetically rearranged viruses through recombination, a process directly associated with replication and the aforementioned DNA polymerase. Orthopoxvirus replication is intimately tethered to high frequencies of homologous recombination between co-infecting viruses, duplicated sequences of the same virus, and plasmid DNA transfected into poxvirus-infected cells. Unfortunately, the effect of these genomic alterations on the cellular context for all poxviruses across the family *Poxviridae* remains elusive. However, emerging sequence data on currently circulating and archived poxviruses, such as the genera orthopoxviruses and capripoxviruses, display a wide degree of divergence. This genetic variability cannot be explained by clonality or genetic drift alone, but are probably a result of significant genomic alterations, such as homologous recombination, gene loss and gain, or gene duplications as the major selection forces acting on viral progeny. The objective of this review is to cross-sectionally overview the currently available findings on natural and laboratory observations of recombination in orthopoxviruses, capripoxviruses, and leporipoxviruses, as well as the possible mechanisms involved. Overall, the reviewed available evidence allows us to conclude that the current state of knowledge is limited in terms of the relevance of genetic variations across even a genus of poxviruses as well as fundamental features governing and precipitating intrinsic gene flow and recombination events.

## Introduction

In contrast to DNA viruses, RNA viruses have evolved a survival strategy involving high mutation rates, large effective population sizes and short generation times to evade and counter the host immunity (Geoghegan and Holmes, 2018). In their turn, large DNA viruses which exhibit low mutation rates, have developed alternative adaptive strategies to increase viral fitness and host range as well as evading the host immune system. Besides accumulating and fixating non-synonymous mutations through positive selection in ORFs of interest, poxviruses frequently employ the strategy of recombination to rapidly generate large genomic variation (Bratke and McLysaght, 2008; Hatcher et al., 2014). Recombination is defined as the process of creating new genomic sequences by combining different gene segments. This process requires the breakage, exchange and rejoining of genome fragments originating from different parental genetic material. The genomes of the resulting recombinant progeny contain different combinations of genetic information acquired from both parental sequences. Recombination is recognized as an important factor during the production of genetic diversity upon which natural selection and evolution can operate (Bujarski, 1999). In virology, recombination is a natural process occurring during replication enabling the repair of damaged replication forks. It is subsequently a pervasive process generating diversity by joining variants originating from either different co-infecting viruses or other replicating genomes within the same host cell. The process ensures the survival of viral progeny by repairing replication errors, as well as creating new opportunities for viruses to overcome selective pressures or adapt to new environments and hosts (Pérez-Losada et al., 2015). For most DNA viruses, recombination is believed to depend upon cellular enzymatic activity, and could be classified as either homologous recombination (general recombination) or non-homologous recombination either by non-homologous end joining or site-specific recombination. Homologous recombination occurs between two DNA sequences that are either identical or share significant similarity in the region/s where crossovers occur. Homologous recombination likely occurs in every DNA-based organism and occurs at a higher frequency compared to non-homologous recombination, in certain organisms such as viruses. DNA viruses mostly utilize homologous (general) recombination machinery available from the host cells, although some viruses encode their own proteins involved in replication and recombination. Non-homologous recombination events have been detected in various classes of single stranded (ss) and double-stranded (ds) RNA and DNA viruses, but the focus of this review will be on homologous recombination in poxviruses (Hristova et al., 2020).

Poxviruses are currently enjoying an increase in research interest, due to the global impact they have on the health of humans and animals (Costello et al., 2022; Rao et al., 2022; Krotova et al., 2022a). They are large DNA containing viruses that include but are not limited to *Orthopoxvirus*, *Parapoxvirus*, *Leporipoxvirus*, and *Capripoxvirus* genera. Numerous outbreaks in both humans and animal populations have been attributed to these viruses in the past, with the human pathogen variola virus (VARV) being successfully eradicated in the previous century (Fenner et al., 1988). This was achieved through vaccination with a related orthopoxvirus, vaccinia virus. However, Damaso et al. (2000) reported a vaccine-related virus variant, circulating in the human and bovine populations of South America called Cantagalo virus (CTGV; Medaglia et al., 2009; José da Silva Domingos et al., 2021). The current epidemiological scenario involving cows and animal handlers, especially milkers, indicates the circulation of various vaccinia virus derivatives in an extensive area of Brazilian territory as well as other South American countries including Argentina, Uruguay, and Colombia (José da Silva Domingos et al., 2021). Such virus variants may have become established in the wild and are maintaining transmission cycles in one or more unknown hosts, prior to adapting as a zoonotic disease (Kroon et al., 2011). Furthermore, monkeypox is continuing to re-emerge in humans at greater frequency (Bunge et al., 2022).

Poxviruses of veterinary importance includes three viruses belonging to the *Capripoxvirus* genus, lumpy skin disease virus (LSDV), sheeppox virus (SPPV) and goatpox virus (GTPV) as well as the myxoma virus (MYXV) from the genus *Leporipoxvirus*. The Capripoxviruses pose a serious threat to the global livestock industry (Tuppurainen et al., 2017), while MYXV is threatening the wild hare populations in Europe. This is due to human attempts to utilize MYXV as a biocontrol measure that has spiraled out of control and led to an increasing arms race between host and poxvirus (Wells et al., 2018).

Importantly, poxvirus species are both phylogenetically different from one another and display variability in host range (McFadden, 2005). Certain viruses including vaccinia have a wide host range, while other poxviruses, such as variola virus, myxoma virus, sheeppox virus, goatpox virus and LSDV, exhibit a restricted host range. This narrow host range could contribute to constraining the virus replication, resulting in lower mutation rates observed within the viral genomes (Hughes et al., 2010; Carroll et al., 2011). The relatively stable genome integrity identified within poxvirus populations, could be attributed to both the high-fidelity polymerase of poxviruses as well as the host restriction stabilizing the virus against further genetic shift through recombination with different poxviruses, capable of co-infecting the same host, limiting these viruses to genetic drift. However, all poxviruses seem to have diverged from a currently unknown common ancestor by adaptive evolution through mechanisms including gene gain and/or loss, horizontal transfer (HT) of host derived genes, as well as recombination between different poxviruses (Gershon and Black, 1988; Hughes and Friedman, 2005). The genomes of poxviruses contain various genes derived from their eukaryotic hosts including transposable elements (Hughes and Friedman, 2005; Filée et al., 2007; Filée, 2009). Many poxvirus genes are orthologs of host genes that seem to have been acquired and “repurposed” to serve other functions (Vallée et al., 2021). The horizontal acquisition of numerous cytokine-like genes and receptors has likely played an important role in helping poxviruses evade host immune responses.

Poxviruses also seem to have changed hosts recurrently during their evolution concomitant with a novel species emergence (Hughes et al., 2010), increasing its ability to transfer sequences among distantly related species. For example, a transposable element related to sequences from snake genomes has been identified in the genome of a taterapox virus isolated from a rodent (Piskurek and Okada, 2007). Another example of foreign retroviral insertions was reported in field and vaccine strains of fowlpox virus (FPV) that carry integrated sequences from avian retrovirus, reticuloendotheliosis virus (Hertig et al., 1997; Umar et al., 2021). These studies indicate, poxviruses may be good HT vectors due to their large genomes and low host and cell specificity, but have a great potential to tolerate genetic insertions.

Demonstrating the actual evolutionary history of various viruses in the field is still lacking, although some work studying MYXV evolution in Australia has provided insights into the process (Kerr et al., 2015). Next generation sequencing (NGS) and third generation sequencing (TGS) technologies have been used to generate complete genome sequences of many poxviruses. This allows for the investigation of molecular evolution and identification of recombination within currently circulating and archived viral genomes. Despite the many publicly available and complete viral genome sequences, elucidating the exact evolutionary pathway involved is challenging and sometimes contradictory conclusions are proposed based on the different methods employed during sequence analysis. One example concerns VARV. Researchers, using archival data on smallpox outbreaks combined with different phylogenetic analysis methods, have obtained contradictory results on the possible time of VARV emergence. Shchelkunov (2009) and concluded that VARV probably separated from a cowpox virus-like agent 3,000–4,000 years before present (YBP). Smallpox became endemic in West Africa in the 14th century AD, where it circulated and evolved in this geographically isolated region. Approximately 300–400 YBP, the West African VARV variant imported into South America diverged into the Alastrim subtype. In contrast, Li et al. (2007) postulated that the two clades separated from the ancestral rodent virus between 16,000 and 68,000 YBP. Clade P-I spread from Asia, between 400 and 1,600 YBP. Clade P-II diverged from an ancestral VARV between 1,400 and 6,300 YBP, followed by subsequent divergence into two subclades at least 800 YBP (Lourie et al., 1975; Li et al., 2007). The evolution into a new species was attributed to multiple deletions in the genome of an ancestral cowpox-like virus (CPXV) and mutations in O1L gene, which allows for efficient replication of VARV in human cells (Smithson et al., 2014; Babkin and Babkina, 2015), providing the virus with its current genetic features. More intriguing findings were extracted from unmineralized skeletons of individuals from the Viking age (1,400 years ago) that had fallen victim to an extinct but distinct variola-like lineage of orthopoxvirus. This extinct lineage forms a sister clade to the currently known variola-like viruses with a divergent pattern of active gene content (Mühlemann et al., 2020). Another ancient VARV obtained from a 17th century Lithuanian mummy demonstrated the same pattern of gene loss as 20th century VARVs, suggesting that the diversification of major viral lineages only occurred within the 18th and 19th centuries (Duggan et al., 2016).

Overall, in this review we summarize the available findings on published evidence of putative mechanisms involved in the observed recombination patterns and gain insights into naturally occurring cases of recombination both natural and experimental in orthopoxviruses, capripoxviruses, and leporipoxviruses.

## Requirements for the generation of recombinant poxviruses

The most recognized modern drivers of pathogen emergence are climate change, environmental destruction leading to increased interactions with new hosts, globalization allowing spread of pathogens to new regions and populations, and interactions with host immune systems. These factors provide a driving force for evolution and adaptation of the pathogen, including mechanisms such as recombination, point mutations in critical genes and the loss or gain of genes (Elde et al., 2012). Recombination enables evolution through the acquisition of new genes that potentially can contribute to novel features such as improved viral replication in a novel host, altered tissue tropism and evasion of the immune system in progeny viruses, which is absent in the parental strains. Even though the primary function of recombination is to ensure viable offspring by assisting with genomic replication, the mechanistic by-product of this process can still serve a future purpose of promoting genetic evolution through fixing selected variants in a given host-associated population (Simon-Loriere and Holmes, 2011).

Intergenic recombination is precipitated when a cell infected with one virus is coinfected/superinfected by another. In order to generate recombinant poxviruses and see them out in the field, several other requirements need to be fulfilled. The first requirement is that two different viruses need to enter the same cell. However, understanding this requires an understanding of how superinfection is governed. In laboratory experiments, homologous recombination has been widely used to disrupt and/or insert genes of interest, to study the functions of specific genes, and to generate recombinant viral vectored vaccine candidates (Wallace et al., 2020; Douglass et al., 2021). Poxviruses are suitable vaccine vectors due to their conserved large genomes, relative safety, and capacity to elicit an adequate immune response (Pastoret and Vanderplasschen, 2003). Examples of recombinant vaccines include the vaccinia rabies vaccine (Pastoret and Brochier, 1996), LSDV based Rift Valley fever virus (RVFV) (Wallace et al., 2020), bovine ephemeral fever (BEFV) (Douglass et al., 2021) and even human immunodeficiency virus (HIV) (Shen et al., 2011; Chapman et al., 2021). The procedures developed for the construction of recombinant vaccinia viruses have been applied to members of other poxvirus genera including avian poxviruses (Boyle and Coupar, 1988) and capripoxviruses (Romero et al., 1993). Although an avian poxvirus is naturally host range restricted, gene expression and protective immunity can be established in non-avian species (Taylor et al., 1988). As nonreplicating vectors, avian poxviruses are exceptionally safe recombinant vaccines.

The second requirement is that the recombinant virus(es) must be able to replicate in the absence of wild-type virus in the absence of complementation. This requires that the disrupted gene be non-essential. In a laboratory setting, this is observed when an essential gene is disrupted using homologous recombination with selection markers. In this case, an essential gene disrupted virus is generated by visualization of a fluorescent protein, however, this virus cannot be plaque purified without the function of the essential gene provided by wild-type virus or through gene complementation *in vitro* (van Diepen et al., 2021).

It is also worth noting that if two nearly identical viruses recombine, it can be difficult, if not impossible, to identify recombinant viruses as the genome sequences will share significant sequence similarities. In this case bioinformatic tools capable of detecting recombination events need to be used, however their description is beyond the scope of this review.

## Mechanisms of recombination

The previously described high recombination rates of poxviruses, allow insights into the recombination process, however these observations have predominantly focused on orthopoxviruses and this can be extrapolated to other poxviruses. Recombination in vaccinia was described through analysis of the marker gene, viral thymidine kinase, during serial passages under different growth conditions (Sam and Dumbell, 1981; Nakano et al., 1982; Weir et al., 1982; Ball, 1987). Under selective conditions, the virus maintained the thymidine kinase insertion, whereas under nonselective conditions the virus deleted it through homologous recombination between the duplicated sequences. Additionally, this study indicated that the viral DNA should go through topological contortion to accomplish homologous recombination for the repeated tandems to come in contact. Another interesting insight of this study was that either DNA replication or the expression of early viral genes, or both, are necessary for intramolecular recombination to occur. These observations were confirmed by an experiment involving temperature-sensitive mutants of vaccinia virus, which demonstrated that for recombination to occur early gene products are required (Merchlinsky, 1989). Since poxvirus DNA replication occurs in the cytoplasm, it follows that recombination should also proceed there.

A study by Evans et al. (1988) provides additional support to indicate that recombination is under viral control and occurs concomitantly with DNA replication. Subsequently the same inextricable link between replication and recombination in poxvirus-infected cells was demonstrated by Willer et al. (1999). The seemingly important DNA replication step is governed by the viral DNA polymerase exhibiting both recombinase and replicase activities (Evans et al., 1988; Merchlinsky, 1989). The combination of these activities in a viral enzyme that is essential for survival argues for recombination being a fundamental, but poorly understood process, that is coupled to poxvirus replication (Colinas et al., 1990). Tandem gene duplication events, arising as part of an adaptation strategy in hostile cellular environments, also require a functional viral DNA polymerase (Slabaugh et al., 1989; Erlandson et al., 2014; Cone et al., 2017). With vaccinia virus, a host immune defense gene knock-out virus underwent transient copy number expansion of another gene in an accordion manner to counteract the host selective pressure. Importantly during the deployment of recurrent duplications, a beneficial mutation was gained in one of the duplicated genes that conferred fitness (Elde et al., 2012). The loci involved in the aforementioned experiment will be discussed in more detail in the next section.

Reversible gene copy number variations as a means of adaptation was also observed at another VACV locus (Cone et al., 2017). These studies shed light on how poxviruses can rapidly adapt to changing circumstances despite low mutation rates, but it is unknown if this is the case for all poxvirus genera. Gene duplications have been described as a common feature of many orthopoxviruses genomes, but this phenomenon has not yet been experimentally observed for other genera (Cameron et al., 1999; Elde et al., 2012; Erlandson et al., 2014). Despite the absence of experimental data, gene repeats are described in various viral genomes, indicating the presence of yet unknown factors that could be acting using a different mechanism than described by Cone et al. (2017) and Elde et al. (2012).

**Figure 1:**
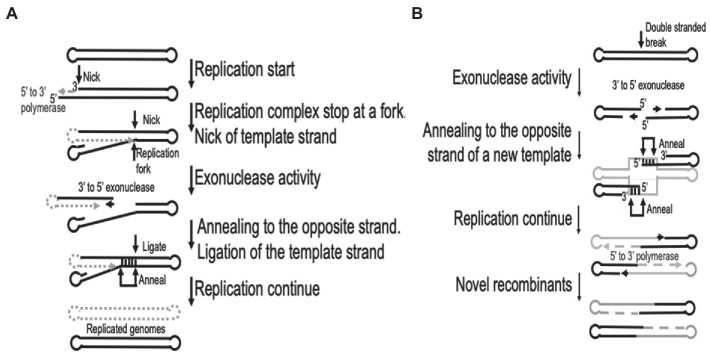
Graphical representation of the recombination processes involved in **(A)**. Replication fork repair and **(B)** double-stranded sequence break repair. This is based on the descriptions by Hamilton et al. (2007). Gray dotted lines represent new synthesis, while gray solid lines indicates a different virus genome in **(B)**.

The processes of replication and recombination in poxviruses are fundamentally linked, either through direct involvement or by utilizing the same enzymes. It has been speculated that recombination could be involved during the initiation of replication, even in the presence of a primase (Fisher et al., 1991; De Silva et al., 2007). Recombination is recruited to resolve arrested replication forks and crucially it is involved during the repair of either single or double-stranded breaks in the template DNA (Michel, 2000). Both replication and recombination require early gene products and monomeric viral genomes as input to produce an abundance of concatemeric heteroduplex DNA molecules (Merchlinsky and Moss, 1989; Fisher et al., 1991). The generation of these concatemer DNA molecules are dependent on a functional virus-encoded DNA polymerase (Evans et al., 1988; Willer et al., 1999). An essential feature of the DNA polymerase enzyme during recombination is the 3′- to 5′-exonuclease activity, which is responsible for initial processing of double-stranded breaks (DBS) into single stranded molecules used during homologous recombination (Yao and Evans, 2003; Gammon and Evans, 2009). The recombination process in vaccinia viruses have been described as using single stranded annealing (SSA) mechanisms to preferentially catalyze the linear-by-linear reactions and thus requiring short (>16 bp) homologous overlaps (Yao and Evans, 2001). This involves the 3′- to 5′-exonuclease catalysis of newly synthesized DNA molecules, interrupted by single stranded nicks or DSBs, resulting in an exposed single stranded 5′ overhang of various lengths exposing sufficient homology to intact template DNA to allow complementary DNA binding (Gammon and Evans, 2009). The processes of replication fork and double-stranded break repair are graphically illustrated in Figure 1, based on descriptions by Hamilton et al. (2007).

Available evidence on orthopoxviruses suggests poxvirus replication occurs in membrane-bound virosomes in the cytoplasm of the host cell, called virus factories. Individual infecting particles generate separate factories shortly after virus infection (Cairns, 1960). These structures are enclosed within the membrane of the endoplasmic reticulum (ER) and gradually increase in size until approximately 6 h post-infection when the virion assembly dissociates the ER (Tolonen et al., 2001). Multiple factories within the same cell could fuse as they increase in size, but the enclosed nature of the factories inhibits the frequent mixing and subsequent recombination of different co-infecting viral particles (Cairns, 1960; Lin and Evans, 2010). This contributes to the high frequency of intragenic recombination (within a single virus factory) during the early stages of virus infection, compared to the increase in intergenic recombination (between co-infecting particles) during the later stages of virus infection when the virosomes fuse or the ER dissociates (Fathi et al., 1991; Paszkowski et al., 2016). It is suggested that despite the same mechanisms involved during intragenic and intergenic recombination, the resulting recombinants displayed a linear relationship between the frequency of recombination events and the distances between mutations located less than 700 bp apart during intragenic recombination. In contrast, intergenic recombination lacks correlation between frequency of events and distance between mutations located between 328 and 10,692 bp apart (Fathi et al., 1991). This is probably due to the high frequency of recombination overshadowing the linkage distributions of markers more than 500 bp apart (Parks et al., 1998).

The genes required to produce all the components involved in viral DNA replication, repair and recombination have been identified. The genomes are large double-stranded linear DNA molecules with terminal repeat regions, that are covalently closed on the ends. DNA replication requires the viral DNA-dependent-DNA-polymerase protein E9, while A20, I3, H6, D4, D5, B1, and A22 are also collectively required to support replication, repair and recombination (Traktman, 1990). The similarity between the structure of the parvovirus replication intermediate and the mature poxvirus genome has led to a model in which replication is initiated at a putative nick proximal to the hairpin (Moyer and Graves, 1981). Strand elongation leads to the formation of concatemers that are later resolved into unit length genomes bearing hairpin ends, by a virus-encoded Holliday junction endonuclease. However, De Silva et al. (2007) later showed that poxviruses encode highly conserved orthologs of D5 protein bearing primase and NTPase activities. Such a primase can function by synthesizing the RNA primers that might be involved in either initiation of replication or lagging-strand DNA synthesis (De Silva et al., 2007). The discovery of more complex head-to-tail molecules seen later during infection, suggested recombination can take place within the terminal repeat regions of the genome (DeLange, 1989).

For interviral recombination to occur, different viruses must infect the same cell. In cell culture, the likelihood of recombination occurring could increase due to an increase in the likelihood of co-infection with different viruses. Furthermore, during the selection of viruses in cell culture the most important factor is the replication fitness of recombinant viruses. This is due to the lack of an adaptive immune system for host defense in cell culture. It was demonstrated in the 1950’s that recombination can occur between two different vaccinia viruses which differed in five phenotypic characteristics; pock morphology, hemagglutinin production, heat resistance, mouse neuropathogenicity, and skin lesions in rabbits. The characterization of recombinant viruses exhibiting different phenotypes demonstrated that 17 of the 149 new viruses displayed a combination of characteristics (Fenner and Comben, 1958). More recently, recombination between two different vaccinia virus strains was assessed using genome sequencing and showed that recombination occurs multiple times per genome. This could generate recombinant viruses with many different gene combinations and expressing many different phenotypes (Qin and Evans, 2014).

It is likely that many different recombinant viruses could be obtained from co-infection of different lumpy skin disease viruses in cell culture. Unfortunately, the phenotypic properties of the recombinant viruses that were derived from a commercial LSDV vaccine (KEVIVAPI), and known to contain sequence elements derived from the LSDV LW-1959/vaccine and Kenyan sheep and goat pox ovine 240 (KSGPO-240) strains, and from GTPV, have not been evaluated (Haegeman et al., 2021). Experimental investigations utilizing three Capripoxviruses with known genomic differences, will help elucidate the frequency and genetic composition of the recombinant viruses in the vaccine as well as subsequent comparison to the circulating recombinant LSDVs observed in the field (Sprygin et al., 2020a,b; Chibssa et al., 2021; Haegeman et al., 2021; Lu et al., 2021). It is likely that multiple different recombinant viruses will be generated with recombination occurring at multiple sites similarly to what has been demonstrated within the vaccinia virus.

## Poxvirus recombinants in the field

Ongoing surveillance of circulating viruses enables not only the early detection of infection in the field, but also tracking of the genetic variants evolving under environmental pressures (Esposito et al., 2006). Orthopoxviruses are the principal focus of research interests in the field of poxviruses due to their impact on human health. During the great wave of smallpox outbreaks in the 19th and 20th centuries, the diagnosis of this disease was based only on clinical symptoms and consequently only archived VARV samples are available which have demonstrated signs of recombination. Since the global eradication of smallpox, experiments into the fundamental aspects of orthopoxvirus biology have been performed under laboratory conditions, which excludes investigations into the naturally occurring variants that emerge in circulating virus populations. In contrast, other poxviruses posing a threat to wildlife and livestock, are currently investigated and their complete genome sequences determined in order to examine evolution (van Schalkwyk et al., 2021).

In recent years, human activities have contributed to the expansion of Capripoxviruses outside of their previously confined regions and humans have also introduced myxoma viruses into Australia as biological agents to control rabbit populations (Kerr, 2012; Tuppurainen et al., 2017). In both of these examples, these viruses are exposed to unusual environmental factors that could impose additional pressure on the evolutionary machinery of the viruses, providing researchers with the unique opportunities to track virus evolution on the genomic level under natural circulation (Krotova et al., 2022a).

### Capripoxviruses

**Figure 2:**
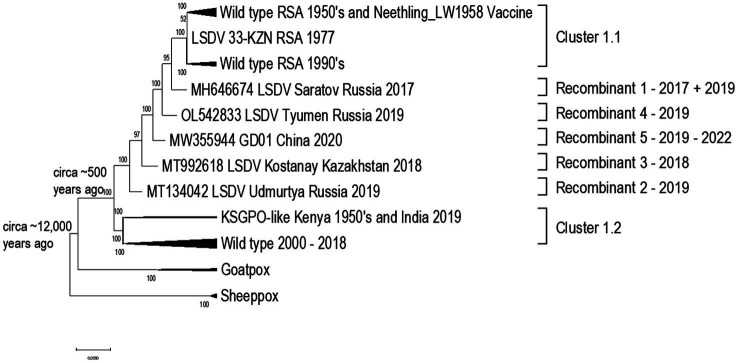
Phylogenetic comparison using a Maximum likelihood tree, under GTR model with 100 Bootstrap iterations, to indicate the relatedness of the capripoxviruses.

Capripoxviruses continue to cause major production losses in endemic countries. LSD was previously confined to Africa, but in 2015 it to Turkey, Europe, Balkans and Russia (Tuppurainen et al., 2017). It has since had a vast and extensive expansion across Russia, Asia and the sub-continent (Sprygin et al., 2019; Chibssa et al., 2021; Haegeman et al., 2021). The three Capripoxviruses of veterinary importance (LSDV, SPPV, and GTPV) arose from a recent common ancestor and divergently evolved with small host ranges. Recent evidence demonstrates that the common ancestor of LSDV is predicted to have emerged around 12,000 years ago (Babkin and Shchelkunov, 2006). Considering the current phylogenetic studies of capripoxviruses, including naturally occurring recombinant LSDV strains that cluster outside of the two established clusters [vaccine/field (cluster 1.1) and field groups (cluster 1.2)], the *capripoxvirus* genomes appear to have evolved through acquiring mutations in intergenic regions and synonymous nucleotide polymorphisms when exposed to host immune systems, or non-synonymous mutations while replicating in cell culture *in vitro* (van Schalkwyk et al., 2021). The phylogenetic relatedness of LSDV strains in comparison to the other Capripoxviruses are summarized in Figure 2.

Gershon et al. (1989) first hypothesized that all *capripoxvirus* species arose from a single ancestor through recombination. This hypothesis was corroborated by analysis of the genetic relatedness between outbreak-associated LSDV samples and the derivative live attenuated vaccine (LAV) strains, using the currently available genome sequences of the *capripoxvirus* members (Sprygin et al., 2018a). Although recombination events between LSD wild-type and vaccine strains were theoretically possible, this has never been observed in South African field isolates, where LAVs have been used for decades (van Schalkwyk et al., 2021). Neither were any recombinants detected in Eastern Europe where mass vaccination campaigns were performed to control or mitigate the concurrent LSD outbreaks, while characterizing the lumpy skin disease viruses in the vaccinated animals (EFSA et al., 2020). Furthermore, observations of recombination have not been observed between LAVs and circulating SPPVs and GTPVs. However, recent outbreaks in Russia demonstrated that recombination events between capripoxviruses were possible and novel recombinant strains were described for the first time (Sprygin et al., 2018a). The first recombinant virus observed was LSDV/Russia/Saratov/2017 where the Neethling vaccine virus (LW-1959/Vaccine) provided the genomic backbone into which genetic segments of another LSDV vaccine (KSGPO-240) were incorporated. Bioinformatic analysis using RDP v4, predicted 27 possible recombination events dispersed across the genome. Not only small genetic segments but also large genomic clusters were exchanged to resemble a wild-type background by reversing truncated genes involved in pathogenesis and host range. This was the first documented recombinant between capripoxviruses to be observed in nature (Sprygin et al., 2018b). As mentioned earlier, recombination events can provide properties unique to the offspring that do not exist in the parental strains which can be unexpected. In a controlled experiment LSDV/Russia/Saratov/2017 was shown to be capable of contact transmission - a feature never before reported for LSDV (Aleksandr et al., 2020).

Moreover, the detection of LSDV/Russia/Saratov/2017 was not an isolated event. Since 2018 recombinant strains have become prevalent in the region and rapidly spread across a wide area including south eastern Asia (Flannery et al., 2021; Tran et al., 2021; Wang et al., 2021). These novel recombinant strains form individual monophyletic groups between the two previously described clusters of LSDVs (Biswas et al., 2020; van Schalkwyk et al., 2020), indicating the origin of their parental strains. Following the description and characterization of LSDV/Russia/Saratov/2017, another recombinant LSDV strain was sequenced. This one (LSDV/Russia/Udmurtiya/2019) caused an outbreak during winter in freezing temperatures. The prevailing climatic conditions during the isolation of LSDV/Russia/Udmurtiya/2019 supports the findings that this recombinant virus can spread in the absence of vectors, unlike classical field isolates (Sprygin et al., 2020a). Udmurtiya/2019 exhibited 24 statistically significant recombination events that were not identical to the 27 detected in Saratov/2017 (Sprygin et al., 2020b). In contrast to the latter, the parental strains are switched in Udmurtiya/2019, i.e., the major parental strain was KSGP, while the minor parental strain was a live attenuated Neethling vaccine. The third recombinant virus from Russia was isolated during an active outbreak in Tyumen in 2019 (Krotova et al., 2022b). These different recombinant viruses shared parents with the other strains from Russia (Krotova et al., 2022a,b). Currently, there are no published descriptions of another novel recombinant isolate (LSDV/Konstanay/Kazakhstan/2018), except for a GenBank entry (MT992618), which exhibits its own unique pattern of predicted recombination events distinct from either LSDV/Russia/Saratov/2017 or LSDV/Russia/Udmurtiya/2019. In 2019, the first outbreak of LSD in the People’s Republic of China (PRC) was reported in Xinjiang province, approximately 20 km from the border with Kazakhstan (Lu et al., 2021). Subsequent determination of the complete genomes from viruses isolated during outbreaks in China and Vietnam indicated that these LSDVs shared a significant percentage of sequence identity among themselves, while forming a unique cluster amongst the recombinant viruses (Flannery et al., 2021; Tran et al., 2021; Wang et al., 2021; Krotova et al., 2022a). These four recombinant strains (LSDV/Russia/Saratov/2017, LSDV/Russia/Udmurtiya/2019, LSDV/Konstanay/Kazakhstan/2018 and LSDV/GD01/China/2019) are genetically unique viruses derived from recombination between vaccine strains LW-1959/vaccine and KSGPO240 (Krotova et al., 2022a). Recombination between LW-1959/vaccine and KSGPO240 is not a new phenomenon, though only through sequencing of partial or complete genomes have recombinants recently been detected. A recent study evaluated archived samples from eastern Africa, prior to lumpy skin disease occurring in the Middle East. This study showed that one field virus collected in Kenya in 2011 (LSDV/Embu/B338/Kenya/2011) displayed mixed genetic features deriving from the vaccine strains LW-1959/vaccine and KSGPO-240. Indicating yet another potential new recombinant strain (Chibssa et al., 2021).

Interestingly, all of the recombinant LSDVs that have been observed in the field thus far, are genetically associated with the vaccine strains LW-1959/vaccine and KSGPO-240. Additionally, the spatial and temporal distribution of these recombinant strains coincide with the use of a contaminated live attenuated vaccine, commercially produced by KEVIVAPI (Kenya; Haegeman et al., 2021). This commercial vaccine has been analyzed, using partial genome sequencing and bioinformatic tools, and this revealed the presence of viral sequences aligning to LW-1959/vaccine, KSGPO-240 and goatpox virus (Haegeman et al., 2021). This indicates the presence of either multiple Capripoxviruses and/or novel recombinant viruses in this vaccine. Despite all the evidence for multiple new recombination events in LSDV, a single lineage has survived selection and established itself in southeast Asia (Tran et al., 2021; Wang et al., 2022). Named after the first detection in China in 2019, the vast expansion of viruses belonging to this Chinese lineage, might be due to either enhanced viral fitness allowing domination over other genetic variants or because it spread into a susceptible cattle population in the absence of competing LSDVs.

Over the last couple of decades, numerous studies have evaluated the transmissibility of LSDV, especially focusing on its vector-borne potential (Sprygin et al., 2019). Interestingly, the role for mechanical transmission with vectors is only shown under laboratory conditions using biting insect vectors to transmit LSDV (Issimov et al., 2021). Originally, LSD outbreaks have predominantly occurred in the rainy summer months associated with the high prevalence of insect vectors (Mafirakureva et al., 2017). The seasonality of the first disease outbreaks in Russia also provides some evidence of the role of vectors in the transmission of the disease (Sprygin et al., 2018a). Unfortunately, no conclusive link could be established between the mechanical transmission in the field and the spread of the virus. Although contact transmission has not been ruled out, it has been considered an inefficient transmission route prior to the arrival of recombinant LSDV (Sprygin et al., 2019). The characteristics enabling the novel recombinant LSDVs to be transmitted more efficiently by contract in the absence of vectors, could possibly explain transmission in months outside the optimal insect vector season (Sprygin et al., 2019). Possessing the phenotypic ability of transmission through both mechanical insect vectors as well as direct/indirect contact, could provide the LSDVs with an additional advantage over the wild-type field isolates. It is evident that the most prevalent route for transmission of LSDV is mechanical transmission *via* insect vectors (Kononov et al., 2019). This is demonstrated by the differences in the spread of sheeppox and goatpox compared to lumpy skin disease (Babiuk et al., 2008). Sheeppox and goatpox are primarily transmitted *via* direct contact and subsequently have restricted spread into new regions in Asia. This is in contrast to LSD that circumvents biosecurity at national borders due to its transmission through insect vectors.

### Leporipoxviruses

A *leporipoxvirus* called myxoma virus (MYXV), might be the best reported example of host-virus coevolution in the field, following a new cross-species introduction. There are two examples of this coevolution on two different continents, Australia and Europe.

The first example is Australia, where MYXV has since 1950 evolved to transmit in a new host species, the European rabbit. This occurred with an experimental field study starting in 1950 in Australia, with the aim to evaluate if MYXV could be used as a biological control tool against European rabbits (*Oryctolagus cuniculus*). This accidentally initiated one of the largest natural experiments in host-pathogen coevolution. The virus strain originally released in Australia, MYXV-SLS (the Brazilian Standard laboratory strain, SLS), is believed to be derived from an isolate first obtained in Brazil in *circa* 1910 (Moses, 1911). Phylogenomic analysis of MYXVs sampled from 1950 to 1999 revealed a gradual genetic drift across all the Australian lineages, revealing a clock-like evolutionary process. In contrast, analysis of 49 newly obtained MYXV genome sequences, isolated in Australia between 2008 and 2017, indicated that MYXV evolution in Australia can be characterized by three lineages, one of which underwent expedited evolution and exhibited a dramatic breakdown of temporal structure. These evolutionary changes could be linked to coevolution between MYXV and the European rabbits in Australia, providing insights into virus adaptation to new hosts and the evolution of virulence. An analysis of epidemiological data revealed that the emergence and establishment of the fast evolving lineage timed with a hemorrhagic disease virus-related shrinkage of wild rabbit populations and prolonged drought, which allowed Kerr et al. (2019) to suggested that the observed punctuated evolutionary event may reflect a change in selection pressures, precipitating virus adaptation to new conditions through genomic changes.

The second example is in Europe, where the virus was introduced in 1952 as a means to control wild rabbit population, again using the Brazilian Standard laboratory strain (MYXV-SLS; Fenner and Fantini, 1999). Unfortunately, the virus rapidly spread to domestic rabbits, and by 1954, 30–40% of the rabbit industry in France had been destroyed (Fenner and Fantini, 1999). The spread was due to a different Brazilian strain, named the Lausanne [lau] strain. It resulted in the spread of MYXV throughout Europe, including the United Kingdom (Wilkinson, 2001). Following the dramatic decline of the wild rabbit population, the fatality rate decreased due to two possible factors; (i) natural selection for slightly attenuated or reduced virulence viruses, or (ii) an increased resistance to myxomatosis in the wild rabbit population (Marshall and Fenner, 1958; Ross and Sanders, 1984; Kerr, 2012). The latter case is known as an arm’s race between the virus and host; i.e. an increase in host resistance leads to a more virulent phenotype of a virus. Although MYXV does not seem to cause significant clinical disease symptoms in the natural cottontail rabbit (*Sylvilagus*) hosts, it has been highly pathogenic to the naive European rabbit (*Oryctolagus*) host, making it a classical example of a pathogen that is highly virulent in a new host species with no history of adaptation.

Several live attenuated vaccines have been developed and used in Europe and Australia, starting with the California MSD strain that has been reported to be less pathogenic than the California MSW strain (Silvers et al., 2006). Saito et al. (1964) developed an MSD-derived strain in California, but it displayed myxomatosis symptoms in rabbits (Saito et al., 1964). Therefore, additional attempts to further attenuate the Saito strain were made and certain of these new LAVs are currently used throughout Europe, for example the Borghi (Jacotot et al., 1967) and MAV strains (Cancellotti, 1985). Moreover, in 1977 Saurat et al., generated a new vaccine strain called MYXV-SG33 by passaging a MYXV isolate obtained from a rabbit killed in the Toulouse area of France in 1973 (Saurat et al., 1978). This LAV MYXV-SG33 as well as Shope fibroma virus have been used as commercial vaccines (Gorski et al., 1994).

**Figure 3:**
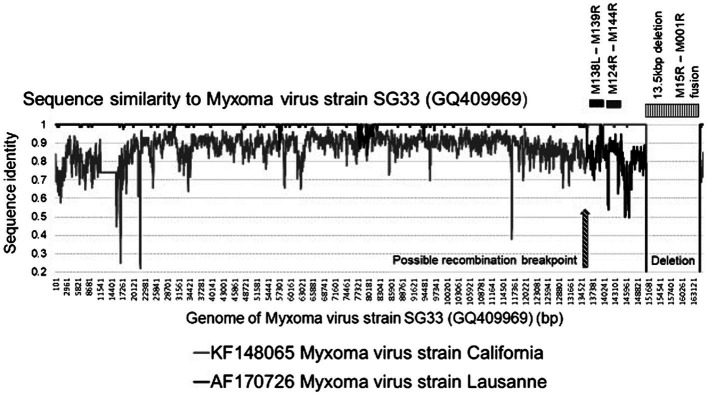
Graphical representation of the sequence identities of the Myxoma virus strains California (KF148065) and Lausanne (AF170726) against the sequence of strain SG33, using a 200 bp window, 20 bp step and Kimura-2 model in SimPlot. The 13 kb deletion in the SG33 genome is indicated as well as the predicted recombination events.

With the development of NGS methods, the full genome sequence and subsequent comparative analysis of MYXV field isolates and vaccine strains has been conducted in several studies to detect recombinants and track the evolution of these different strains. Guerin et al. (1998) indicated that the LAV MYXV-SG33 strain has a large deletion in the right terminal region of the genome (Guerin et al., 1998), while later partial sequencing of 15 genomic location (spanning 35 genes) of the same isolate conducted by Cavadini et al. (2010) indicated a high percentage sequence identity (97–100%) to the Lausanne isolate. But when analyzing genes M138L-M139R and M142R-M144R the percentage sequence similarity decreased to 84 and 89%, respectively, (Guerin et al., 1998). Nevertheless, M138L-M139R showed 100% identity with the MYXV-MSD sequence (Cavadini et al., 2010). The full analysis of the MYXV-SG33 genome was performed by Camus-Bouclainville et al. (2011) and they confirmed the deletion in the right region of the genome with evidence of a field recombination between wild-type and vaccine strains (Figure 3).

Results showed that the SG33 genome is 13.5 kbp shorter than that of Lausanne, confirming the deletion in the right region of the genome, resulting in the absence of 13 genes and the in-frame fusion of the truncated M151R and M001R open reading frames (ORF’s; Camus-Bouclainville et al., 2011). Moreover, considering the fact that the California isolate has never been handled in the laboratory of Toulouse, where SG33 was obtained, and MSD was used as a vaccine for years, demonstrated that this strain is not completely attenuated. It can still disseminate and infect wild rabbits, where Lausanne-like strains were circulating especially in the Toulouse area. The authors suggest that the only possible explanation for this dual origin of SG33 is that the isolate used to generate it was itself a product of a field recombination between a virulent South America strain and a vaccine California strain (Camus-Bouclainville et al., 2011).

However, this is not the first study to demonstrate field recombination in leporipoxviruses. Another study established that Malignant rabbit virus (MRV) is the result of recombination between MYXV and Shope fibroma virus (SFV; Upton et al., 1988). The authors indicated recombination junctions at both the left and right terminuses of the MRV genome. Recombination at the left terminus derived T5, T6, T7, and T8 from SFV, while at the right terminus upstream from the SFGF gene of MRV creates an in-frame fusion in ORF T11-R in which the N-terminal amino acids are derived from MXYV and the remainder from SFV (Upton et al., 1988).

The most recent study analyzing probable recombination in the genome of MYXV was performed in 2019 by Agueda-Pinto et al., where the whole genome sequencing of a MYXV Toledo isolate was performed and genetically characterized (Águeda-Pinto et al., 2019). The analysis into the genetic background of the viral agent was motivated by an unprecedented death rate in wild hares, the brown hare (*Lepus europaeus*) and the Iberian hare (*Lepus granatensis*) with clinical signs suggestive of myxomatosis. Since the rabbit (*Oryctolagus cuniculus)* was the only target host of the myxoma virus, the observed findings strongly argued for host range expansion within the family *Leporidae* due to genome alterations. The virus was isolated from a dead female wild hare and designated MYXV-Tol. Analyses of the MYXV-Tol complete genome sequence revealed an insertion of ~2,800 bp in the left terminal region of the genome in comparison to the sequence of MYXV-Lau. This new recombinant region disrupted the M009L gene of MYXV-Lau, without completely deleting the gene, instead regions of the M009L gene were present and flanking the new 2,8 kbp region. Moreover, this recombinant insertion contains at least four ORFs that are predicted to encode four viral proteins homologous, but not identical, to the poxvirus gene families exemplified by the M060R, M061R, M064R, and M065R genes from MYXV. The complete genome of MYXV-Lau contains a locus at ~57,500 bp that includes a set of six ORFs (M060R to M065R) conserved in all MYXVs, including MYXV-Tol (Cameron et al., 1999; Kerr and McFadden, 2002). The four predicted ORFs inserted into the new recombinant region of MYXV-Tol, are similar to the region around ~57,500 bp, with the exception of two deleted ORFs (M062R and M063R) situated on the negative strand of the genome. This virus gene arrangement was limited to ORFs encoded by the genomes of capripoxviruses, cervidpoxviruses, suipoxviruses, yatapoxviruses, and three unclassified poxviruses (BeAn 58,058 virus, cotia virus, and eptesipoxvirus; sharing 69–73% nucleotide identity. These results suggest that the recombinant region was derived from a new, still unreported or unidentified poxvirus, which shares a common ancestral origin with capripoxviruses, cervidpoxviruses, suipoxviruses, yatapoxviruses, and the three unclassified poxviruses (BeAn 58,058 virus, cotia virus, and eptesipoxvirus).

## Loci targeted by recombination

Recombination has been associated in selecting novel phenotypes, including temperature-sensitive mutants, plaque size, pock morphology, virulence and the ability to evade the host immune system (Bedson and Dumbell, 1964; Dumbell and Bedson, 1964; Elde et al., 2012). With the onset of NGS, identifying the genomic regions involved during phenotype selection due to recombination, have been elucidated (Esposito et al., 2006; Qin et al., 2011, 2013; Qin and Evans, 2014). Examples of these genomic modifications include large deletions in the left terminal repeat regions associated with the small plaque forming phenotype of some VACV TianTan sub-clones. Similarly, the increase in variation of the number of nucleotide repeats, often resulted in additional amino acids included into the reading frame of genes in the VACV Dryvax vaccine, these repeat expansions often did not alter the reading frames as many were multiples of three (Qin et al., 2011, 2013). The number of Vaccinia virus K3L gene copies can increase by 15 to 20 copies to counteract the host immune’s antiviral protein kinase R (PKR). Each copy increases the probability of obtaining novel non-synonymous substitutions, prior to decreasing the number of gene copies and subsequently fixating novel substitutes in the viral population (Qin and Evans, 2014). This gene accordion strategy is a fast and effective method to acquire single nucleotide polymorphisms (SNPs) capable of changing the phenotype in order for the virus to adapt, survive or out-compete external competition or immune pressure (Roth and Andersson, 2012).

Genome analysis of multiple VACV TianTan and Dryvax hybrids provided significant insights into the ORFs affected by recombination events, either by sequencing individual clones of a quasispecies population obtained from each of the vaccine strains or by performing co-infection experiments using clones derived from both of the vaccines. Differences between the progeny VACV hybrid clones were due to indels, which resulted in both frameshift as well as in-frame changes to the coding regions, large deletions removing up to 11,7 kb regions of the telomeres and multiple SNPs (Qin et al., 2011, 2013). Each of the genomic differences observed in the offspring could be assigned to either one of the parental sequences, but in different rearrangements (Qin et al., 2011, 2013). The central core region of the VACV genome contained the majority of the conserved or nearly identical ORFs. This is in contrast to the highly variable telomere regions where only nine ORFs were unchanged between all the Dryvax clones (Qin et al., 2011). If we compare the recombinant progeny obtained from co-infection of the two VACV vaccine strains with the recombinant LSDVs identified in the field, a number of interesting similarities and differences come to light. LSDVs are clustered into two distinct clusters (Cluster 1.1 and 1.2), that constitutes the parental strains for the novel recombinant viruses observed between 2011 and 2021 (Sprygin et al., 2018b, 2020b; Flannery et al., 2021; van Schalkwyk et al., 2021). In contrast to the VACV clones, differences between LSDV LW-1959/vaccine and KSGPO-240 involved nearly every ORF, with the exception of seven (LW004, LW014, LW016, LW044, LW104, LW106 and LW120). These conserved LSDV ORFs predominantly encode proteins involved in intracellular mature virions (IMV) membrane formation (LW104, LW106 and LW120), yet the elf-2-alpha structural PKR inhibitor (similar to VACV K3L) LW014 and EGF-like growth factor (LW016) are also conserved. Gene duplication and deletion similar to the accordion effect observed in VACV K3L, have not been described in LSDV LW014 (Elde et al., 2012; van Schalkwyk et al., 2020). There were ~2,184 nt differences described over the complete genomes of LSDV vaccine strains LW-1959/vaccine and KSGPO-240, with certain SNPs involved in altering the reading frame of 10 ORFs (Kara et al., 2003; van Schalkwyk et al., 2021). This is in contrast to the 73 and 44 ORFs with alternative reading frames, respectively described for the DryVax and TiaTan VACV vaccine clones (Qin et al., 2011, 2013). Since frameshift mutations are responsible for rapid adaptations to novel phenotypes, it is not surprising that significant overlap exists between the affected predicted proteins in LSDV and VACV (Senkevich et al., 2020). The predicted proteins with alternative reading frames are listed in Table 1.

**Table 1:** Predicted proteins displaying differences due to frameshift mutations in both LSDV and VACV (Kara et al., 2003; Qin et al., 2011, 2013; Senkevich et al., 2020; van Schalkwyk et al., 2021).

Function predicted for the affected proteins	Open reading frames in the LSDV genomes	Open reading frames in the VACV genomes
IL-receptor like protein	LW013	VACV_DVX_019; VACV_DVX_032; VACV_DVX_033; VACV_DVX_209
Kelch-like protein	LW019; LW144	VACV_DVX_052; VACV_DVX_203; VACV_DVX_032; VACV_DVX_214; VACV_DVX_215; VACV_DVX_216
RhoA signal inhibitor (F11)	LW026	VACV_DVX_060
RNA decapping enzyme	LW086; LW087	Cop-D10R
Holliday junction	LW114*	VACV_DVX_153*; Cop-A22R
Unknown (B22R)	LW134	VACV_DVX_009

* An essential gene for vaccinia virus and LSDV, thus the frame shift mutation extends the predicted protein in LSDV.

**Figure 4:**
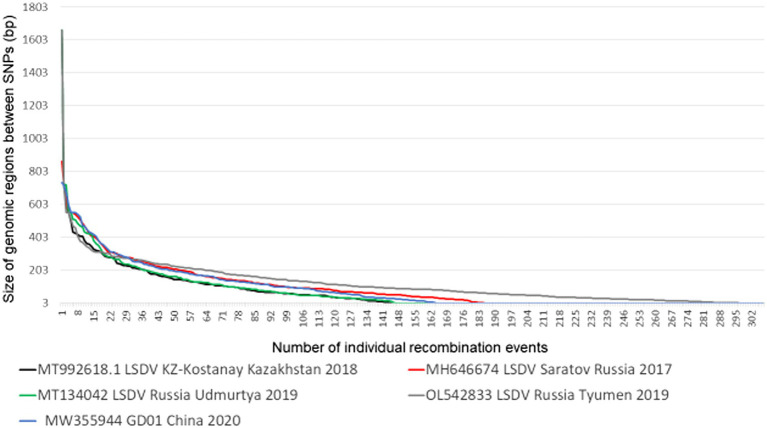
The length of genomic conversion tracts observed in the five novel recombinant LSDV genomes (Picture from Krotova et al., 2022a).

In contrast to both the VACV Dryvax and TianTan hybrid clones, LSDVs do not have large deletions in the terminal regions, differentiating between isolates belonging to either cluster 1.1, 1.2 or the novel recombinant strains. As mentioned previously, the latter are thought to have originated during the production of a contaminated vaccine, where vaccine strains LW-1959/vaccine, KSGPO-240 and goatpox virus were co-infected in cell culture and administered to cattle (Haegeman et al., 2021). This co-infection of multiple LSDV strains are represented by five monophyletic novel recombinant groups, four are phylogenetically represented by a single complete genome sequence (Russia/Saratov/2017; Russia/Udmurtya/2019; Russia/Tyumen/2019 and Kostany/Kazakhstan/2018), while the fifth group currently have nine complete genomes (GD01/China/2020; HongKong/2020, Taiwan/2020, two from Russia/2020 and four from Vietnam/2020) (Krotova et al., 2022a). Similar to the genomic mosaic patterns observed during the co-infection of VACV TianTan (clone TP05) and Dryvax (clone DPP17), the novel recombinant LSDV strains produced a unique insight into the recombination frequency and loci selection of these poxviruses (Qin and Evans, 2014). This co-infection experiment compared clones derived from an infection using a multiplicity of infection (MOI) of 0.02 over five passages (DTM - Dryvax-TianTan mixture) as well as a MOI of 10 over 3 passages (DTH - Dryvax-TianTan high). Of the new hybrid clones, 16 DTM and 15 DTH clones were subjected to full genome sequencing of the SNPs were assigned to either of the parental strains. A recombination event was defined once a SNP belonging to one parent was followed by an SNP identical to the other parent. Using the average 30 exchanges within the DTM clones and 18 in the DTH genomes, the number of crossover events were between 14 to 44 in DTM and 0 to 38 in the DTH genomes. Based on the ~1,400 bp differences between VACV TP05 and DPP17 spanning the 200kbp genome, the frequency of 1 SNP per 140 bp, produced a calculated average conversion tract of 12kbp in length (Qin and Evans, 2014). In comparison, LW-1959/vaccine and KSGPO-240 have ~2,200 bp differences and under the same criteria the average number of crossover events was 196, considering the 10 recombinant genome sequences. This resulted in an average of 1 SNP per 69 bp and an average conversion tract of 150 bp (Figure 4).

**Figure 5:**
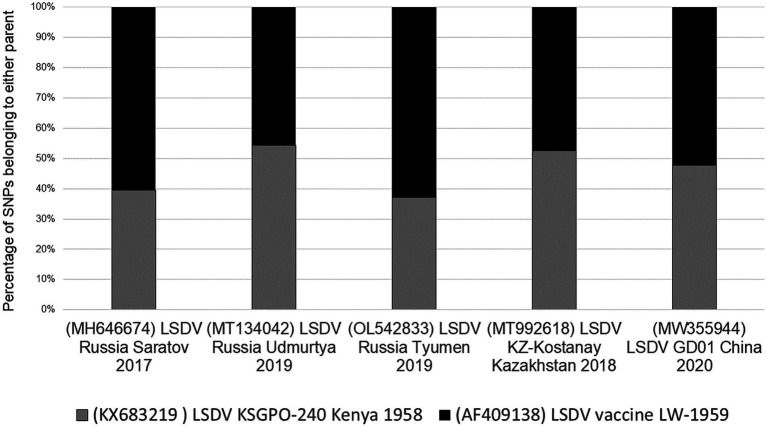
Graphical representation of the percentage SNPs identical to each of the parental sequences for LSDV/Russia/Saratov/2017, LSDV/Russia/Udmurtya/2019, LSDV/Russia/Tyumen/2019, LSDV Kostanay/Kazakhstan/2018 and LSDV/GD01/China/2020 (Picture from Krotova et al., 2022a).

Unlike the selective bias of DTM VACV clones, where only 19% of the genome represented the parental DPP17 genome, both the DTH and LSDV novel recombinants displayed an equal distribution of parental sequences. The novel LSDV recombinants differ between 38 and 62% of parental SNPs with LSDV/GD01/China/2020 strain at an almost equal distribution (Figure 5).

**Figure 6:**
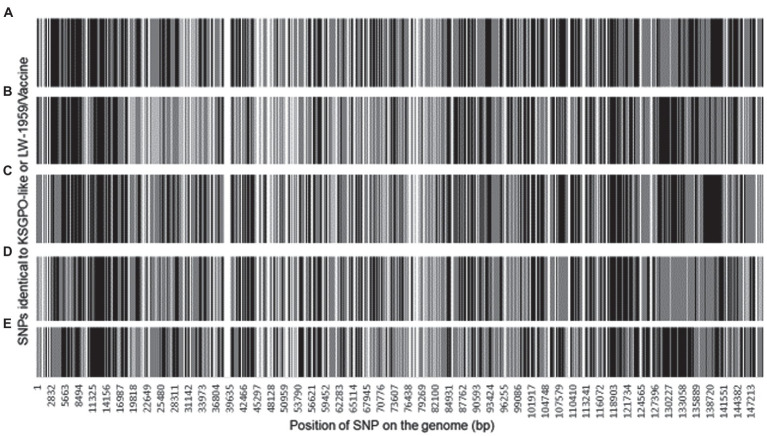
Graphical representation of the genomic position of each SNPs identical to LSDV/KSGPO-240/Kenya/1958 (gray) or LSDV/LW-1959/Vaccine (black), in the genome of **(A)** LSDV/Russia/Saratov/2017; **(B)** LSDV/Russia/Udmurtya/2019; **(C)** LSDV/Russia/Tyumen/2019; **(D)** LSDV Kostanay/Kazakhstan/2018; **(E)** LSDV/GD01/China/2020 (Picture from Krotova et al., 2022a).

The ~2, 200 SNPs are located throughout the LSDV genomes affecting 148 of the ORFs. A total of 1856 SNPs are located in ORFs and could be classified as 1,258 synonymous SNPs and 588 non-synonymous, while the remaining 328 are located in intergenic regions (IGR). The SNPs assigned as either identical to LSDV/KSGPO-240/Kenya/1958 (gray) or LSDV/LW-1959/Vaccine (black) are presented in Figure 6.

The percentage of transition mutations in LSDV parental sequences was 65%, which is comparable with the 72% of SNPs identified in the VACV TP05 and DPP17 clones (Qin and Evans, 2014). Based on the experimental studies performed in VACV, the high number of crossover events in the novel LSDV recombinant strains are indicative of a high passage history during the co-infection of LW-1959/vaccine and KSGPO-240.

**Figure 7:**
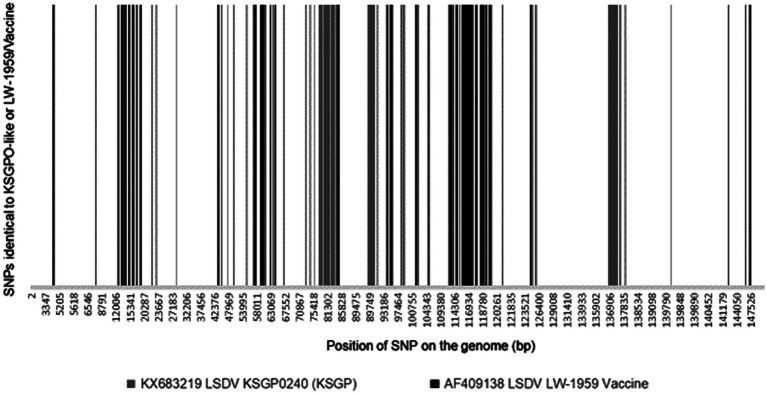
Graphical representation of the genomic positions under selection for either KSGPO-240 (gray) or LW-1959 (black) polymorphisms (Picture from Krotova et al., 2022a).

Recombination and the generation of novel recombinants have been associated with accumulation of various phenotypic advantages (Bedson and Dumbell, 1964; Dumbell and Bedson, 1964; Elde et al., 2012). The complete extent of the phenotypic advantage/s obtained by the novel LSDV recombinant strains have not yet been determined. Experimentally, the strains have been implicated in transmission during the absence of insect vectors (Kononov et al., 2019) and an increase in growth rate during culturing on primary cells as well as aggressive growth in cattle (Kononova et al., 2021). Observations from the field have associated these viruses with rapid spread across borders similarly to the previous spread of classical LSDVs, however under vastly different environmental conditions than previously described. These included Russian winters, which are associated with the complete absence of all insect vectors (Sprygin et al., 2020a,b). It was therefore imperative to identify the possible genomic loci associated with the novel phenotypes. Since the novel recombinant LSDV strains displayed a mosaic of short conversion tracts, the SNPs identical to all the novel strains, as well as the ORFs they are located in were identified (Krotova et al., 2022a). A total of 306 SNPs were identified with 113 identical to KSGPO-240 and 193 identical to LW-1959/Vaccine. These SNPs clustered into 63 ORFs with 198 synonymous and 86 non-synonymous SNPs, while 22 were in IGRs (Figure 7).

The majority of the ORFs under selection were involved in either transcription, replication or virion formation (Krotova et al., 2022a). The position of amino acid exchanges as well as the function of each of the predicted proteins are indicated in Supplementary Table 1.

## Conclusion

Recombination plays an important role in the evolution of viruses including poxviruses. High-throughput sequencing of additional poxvirus genomes is contributing to the elucidation of poxvirus molecular evolution from the fundamental perspective. The apparent low mutation rates are offset by high recombination potential under selective conditions manifested by horizontal gene transfer, gene gain and loss and homologous recombination. The orthopoxviruses have enjoyed the majority of the research attention, since some of its members are deadly human pathogens. In contrast, other members of the Poxviridae family have only recently been gaining research focus, due to their expanded global distribution and a high impact on the economy. Owing to the advent of molecular tools, numerous studies have described novel poxviruses subjected to significant genetic changes and all of these novel viruses were associated with potentially new virulent phenotypes. The unprecedented spread of LSDV from Africa into Europe, Russia and Asia, with particular emphasis on the novel recombinant LSDVs, emphasizes our current lack of knowledge with respect to capripoxviruses. The current state of knowledge is limited in terms of the relevance of genetic variations across even a genus of poxviruses as well as fundamental features governing and precipitating intrinsic gene flow and recombination events. Understanding these mechanisms provides tools for harnessing evolution to the benefits of humankind. The increased amount of genome information available together with improvement of tools for analysis will allow for detailed understanding of the evolutionary history of poxviruses and monitoring of adaptation-driven genetic alterations in poxviral genomes in the field.

## Author contributions

All authors listed have made a substantial, direct, and intellectual contribution to the work and approved it for publication.

## Funding

This work was supported by the grant no. 075-15-2021-1054 from the Ministry of Education and Science of Russia to implement objectives of the Federal Scientific and Technical Program for the Development of genetic technologies during 2019–2027.

## Conflict of interest

The authors declare that the research was conducted in the absence of any commercial or financial relationships that could be construed as a potential conflict of interest.

## Publisher’s note

All claims expressed in this article are solely those of the authors and do not necessarily represent those of their affiliated organizations, or those of the publisher, the editors and the reviewers. Any product that may be evaluated in this article, or claim that may be made by its manufacturer, is not guaranteed or endorsed by the publisher.
