# Supplementary material for: Capripoxviruses, leporipoxviruses, and orthopoxviruses: Occurrences of recombination
Source: Front Microbiol. 2022 Oct 6;13:978829. doi: 10.3389/fmicb.2022.978829 (PMC9584655; doi:10.3389/fmicb.2022.978829)
Supplement: Supplementary file 1 [file Table_1.docx]

**List of Abbreviations:**

| Abbreviation | Abbreviated Term |
| --- | --- |
| BEFV | Bovine ephemeral fever |
| CTGV | Cantagalo virus |
| CPXV | Cowpox-like virus |
| DBS | Double stranded breaks |
| DTH | Dryvax-TianTan high |
| DTM | Dryvax-TianTan mixture |
| ER | Endoplasmic reticulum |
| FPV | Fowlpox virus |
| GTPV | Goatpox virus |
| HT | Horizontal transfer |
| HIV | Human immunodeficiency virus |
| IGR | Intergenic region |
| IMV | Intracellular mature virions |
| KSGPO-240 | Kenyan sheep and goat pox ovine 240 |
| LAV | Live attenuated vaccine |
| LSD | Lumpy skin disease |
| LSDV | Lumpy skin disease virus |
| MRV | Malignant rabbit virus |
| MOI | Multiplicity of infection |
| MYXV | Myxoma virus |
| MYXV-SLS | Myxoma virus, Brazilian Standard laboratory strain |
| LW-1959/Vaccine | Neethling vaccine virus |
| NGS | Next generation sequencing |
| ORF | Open reading frame |
| PRC | People’s Republic of China |
| PKR | Protein Kinase R |
| RVFV | Rift Valley fever virus |
| SPPV | Sheeppox virus |
| SFV | Shope fibroma virus |
| SNP | Single nucleotide polymorphism |
| SSA | Single stranded annealing |
| TGS | Third Generation sequencing |
| VARV | Variola virus |
| YBP | Years before present |
